# Unravelling the enigma of siRNA and aptamer mediated therapies against pancreatic cancer

**DOI:** 10.1186/s12943-022-01696-5

**Published:** 2023-01-12

**Authors:** Zhe Liu, Neha Parveen, Urushi Rehman, Aisha Aziz, Afsana Sheikh, Mohammed A. S. Abourehab, Wei Guo, Junhao Huang, Zhenning Wang, Prashant Kesharwani

**Affiliations:** 1 Department of Pancreatic-Biliary Surgery, The First Hospital of China Medical University, Shenyang, China; 2 Department of Pharmaceutics, School of Pharmaceutical Education and Research, Jamia Hamdard, New Delhi, 110062 India; 3 Department of Pharmaceutics, College of Pharmacy, Umm Al-Qura University, Makkah, 21955 Saudi Arabia; 4 Department of Surgical Oncology and General Surgery, The First Hospital of China Medical University, 155N. Nanjing Street, Shenyang, 110001 Liaoning China; 5 Key Laboratory of Precision Diagnosis and Treatment of Gastrointestinal Tumors, Ministry of Education, China Medical University, Shenyang, 110122 Liaoning China; 6 Institute of Health Sciences, China Medical University, Shenyang, 110122 Liaoning China; 7 Center for Transdisciplinary Research, Department Of Pharmacology, Saveetha Dental College, Saveetha Institute of Medical and Technical Science, Chennai, India

**Keywords:** siRNA, Aptamers, Nanoparticles, Combination therapy, Chemotherapeutic, Pancreatic cancer

## Abstract

Pancreatic cancer (PC) is a fatal disease that has a poor 5-year survival rate. The poor prognosis can be attributed to both troublesome detections at the initial stage, which makes the majority of the treatment options largely unsuccessful and leads to extensive metastasis, as well as to its distinct pathophysiological characteristics, such as rich desmoplastic tumours bounded by dysplastic and hypo perfused vessels restricting the mobility of therapeutic agents. Continued attempts have been made to utilise innovative measures for battling PC to increase the therapeutic effectiveness of therapies and overcome their cytotoxicity. Combined cancer targeting and gene silencing approach has shown improved outcomes in patients’ survival rates and quality of life, offering a potential solution to therapeutic complications. It particularly targets various barriers to alleviate delivery problems and diminish tumour recurrence and metastasis. While aptamers, a type of single-stranded nucleic acids with strong binding affinity and specificity to target molecules, have recently surfaced as a viable PC strategy, siRNA can interfere with the expression of certain genes. By concurrently suppressing genes and boosting targeted approach, the cocktail of siRNA/Aptamer and other therapeutic drugs can circumvent the multi-drug resistance phenomena. Additionally, combination therapy with additive or synergistic effects can considerably increase the therapeutic efficacy of anti-cancer medications. This study outlines the primary difficulties in treating PC, along with recent developments in siRNA/Aptamer mediated drug delivery to solve the major hiccup of oncology field.

**Graphical Abstract:**

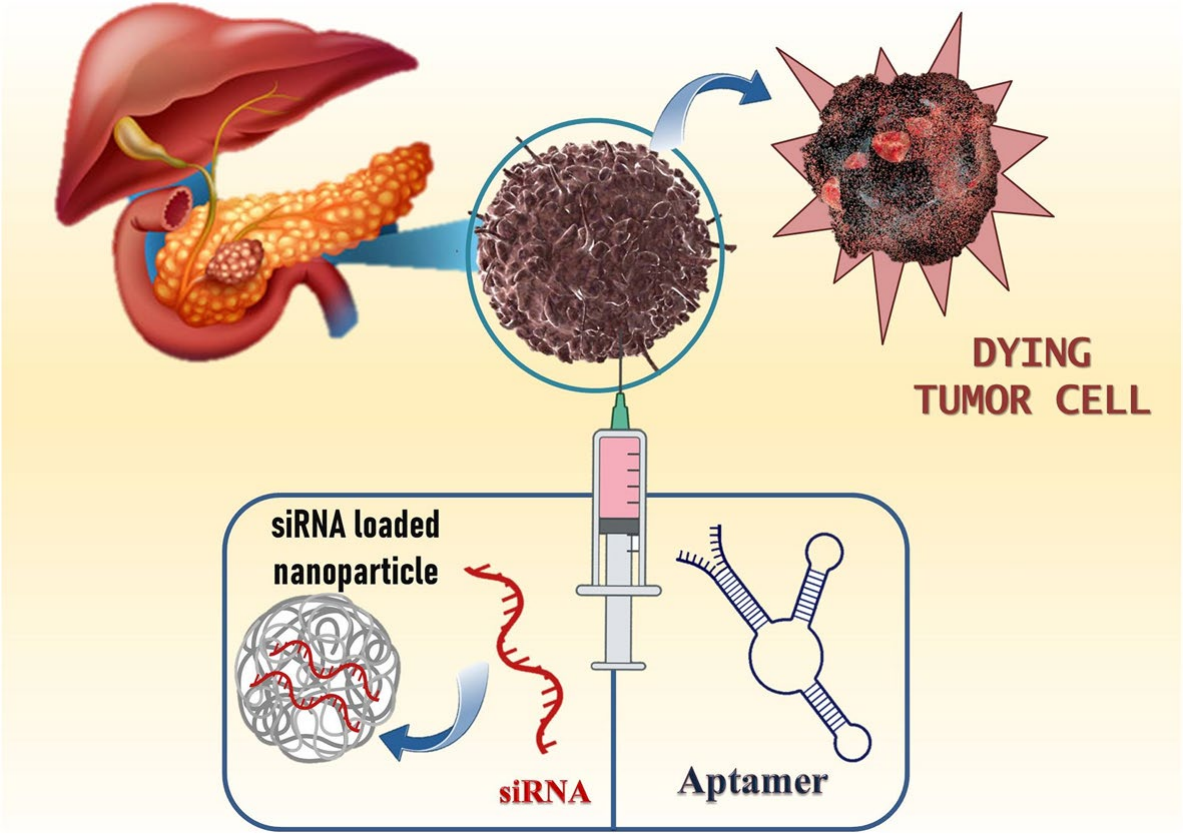

## Introduction

Cancer kills around 8.2 million people worldwide, with those statistics expected to soar up to 12 million by 2030 [1]. Pancreatic cancer (PC) is considered one of the most lethal tumours having an overall survival rate of 5 years less than 9% [2]. It is ranked as the fourth major cause for cancer-related fatalities worldwide [3–10]. The global incidence is at 1–10 occurrences per 1 lakh people, with incidence usually higher in men in developed countries [11–17]. It is found to be more prevalent in the elderly between 60 and 80 years [18]. Environmental and heritable factors are contributing to the development of PC [19] with obesity [18], diabetes [18], smoking [18], chronic pancreatitis, a diet high in sugar and fat content and genetic variables have all been shown to induce PC most commonly [20]. It remains one of the most difficult cancers to treat, with a poor prognosis and fewer therapy options available [21] because it is typically detected at later stages [22]. It is explained by the fact that patients in the initial stages of PC generally show few or ambiguous symptoms which leads to diagnostic challenges [23]. Patients in advanced stages may experience nausea, vomiting, loss in weight unexpectedly, abdominal discomfort, and heartburn [24]. Poor prognosis is partly attributed to fast metastasis, which results in an accelerated course of the disease combined with a lack of effective systemic treatments [25]. PC treatment options available are surgery, chemotherapy and radiation [23]. However, the present treatment options did not show significant improvements in overall survival rates over the last few years, as PC is characterised by complex genetic mutations as well as the growth of compact tumour microenvironment resulting in poor survival of approximately 15–25% of patients who underwent with surgery [26]. Due to the high resistance and toxicity associated with conventional treatments for PC, the development of alternative therapies for PC has been encouraged. Lately, molecular drugs with targeted delivery have demonstrated significant potential. These types of drugs are highly selective in nature, overt the toxic effects but are also restricted to treating patients that are selected based on their genetic makeup of the cancer phenotype [27]. siRNA is double-stranded RNA with a length of 19–23 base pairs that can specifically silence the gene expression of certain genes. But due to its high molecular weight, hydrophilic nature, and the surface negative charge; its entry gets restricted inside the cell. Furthermore, it is susceptible to serum degradation and rapid clearance on administration. Thus nanoparticles are required as carriers for their efficient delivery to cells [28–36]. On the other hand, Aptamers represents single-stranded RNA or DNA with length ranging from 25 to 60 nucleotides. They acquire certain shapes to attach themselves to the target site with high selectivity and affinity. Aptamers have recently been used in the treatment of PC due to their advantageous properties such as good targeting efficiency, thermal stability, and biocompatibility [37–42]. As cancer therapy often involves using drugs in combinations to reduce adverse effects, dose and overcome drug resistance [43], thus combinatorial approach using chemotherapeutics and molecular targeted agents such as siRNA and aptamers is an attractive option. Metabolomics is a technique which seeks to characterise those organic compounds produced by biological processes, that helps increasing our comprehension of cellular biology. The study of metabolites and fatty acids in their own native spatial space, in specific, has been at the frontline of metabolomics and lipidomics investigation [44]. The location and chemistry of small molecules involved in metabolic phenotypes, defence molecules, and chemical interactions in natural communities are described in spatial metabolomics [45]. Molecular processes of each tissue type vary, providing a novel clinical insight into the cancer cells’ molecular chemistry compared to the normal epithelium [46]. The molecular differences and inherent functions among the tissue components results in failure of bulk measurement based methodological approach. This causes mere average measurements and insignificant information. Metabolomic technologies clears that neoplastic cells inherit some metabolic traits for their growth and mediating the cascade of metastasis. Augmented instrumentational techniques made it possible to detect the metabolic profile of numerous cancer cells. To observe the complete picture of biological tissues and vital organs, research scientists are now approaching towards spatial metabolomics for the investigation of biomarkers. With a view to discover cancer tissue relevant metabolic signatures, Zang et al., mapped in vitro model of a high spatially resolved metabolomics combined with a multicellular tumor spheroid (MCTS) with matrix-assisted laser desorption/ionization mass spectrometry imaging (MALDI-MSI). The combined analysis of metabolomic data identified the vital and functional metabolic signatures of oesophageal cancer including fatty acid metabolism, phosphatidylethanolamine and de novo synthesis phosphatidylcholine. The combined approach gave a real-world relevance by identifying the abnormal expression of metabolic enzymes such as choline kinase (CHKA), Glutaminase (GLS), cytosolic phospholipase A2 (cPLA2) and Fatty acid synthase (FASN) [47]. Luo et al., extracted a dozen of cancerous lesions from the PC patients along with paired normal tissues to identify the biomarkers associated with PC cells proliferation and metastasis [48]. Application of metabolomic tools to use siRNA/aptamer in a combination of chemotherapeutic agents has been proven to improve therapeutic outcomes by boosting the sensitivity of cancer cells toward chemotherapeutics or by functioning synergistically [49]. This review introduces the conventional therapies for PC, as well as the complications associated with them. Most importantly, it is focused on the combinatorial approach of chemotherapeutics and siRNA/Aptamer employed for treating PC as well as future considerations.

## Pathogenesis of pancreatic cancer

**Fig. 1:**
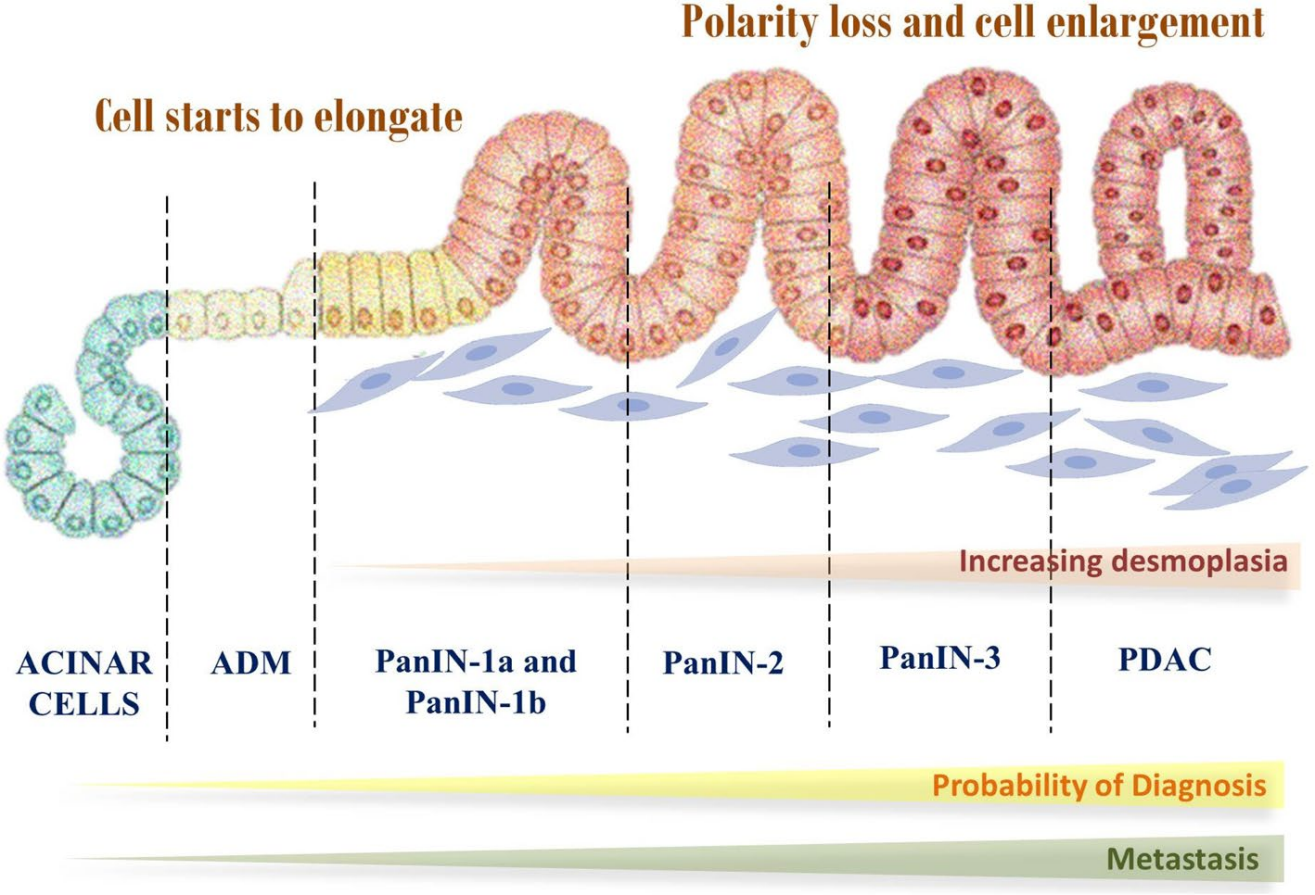
Development and progression of the pancreatic cancer

The pathogenesis of PC follows stepwise mutations, going from the normal mucosa to particular benign tumours, then to cancer. The most well-defined antecedents include mucinous cystic neoplasms, intra-ductal papillary mucinous neoplasms, and pancreatic intraepithelial neoplasia (Fig. 1) [50].

PanIN is a microscopic lesion in the tiny pancreatic ducts that is not invasive (not more than 5 mm). It is suspected to play a part in developing localised pancreatitis, giving rise to a cycle of epithelial injury and repair, which furthers the neoplastic process. Molecular level studies show that PanIN is the most common precursor and these lesions have genetic abnormalities [50]. An effective and beneficial technique to diagnose ductal adenocarcinoma early is PanIN detection; although, because PanINs are tiny tumours with a diameter lesser than 5 mm and tend to originate in branching peripheral ducts, their detection can be challenging. Another form of macroscopic non-invasive lesions are cysts. The dilated ducts and cysts can be detected via radiology; for example, during a radiological scan of the pancreas, a cystic precursor lesion may be discovered. Such unanticipated findings are called pancreatic incidentalomas. The cystic precursors are further divided into MCNs and IPMNs [51].

Intra-ductal papillary mucinous neoplasm (IPMNs) are lesions which are responsible for casuing radiographically visible pancreatic ductal dilatation, that primarily affect the primary pancreatic ducts, the secondary ducts, or either both kinds of ducts [52]. IPMNs and PanINs share many molecular aberrations, although they are frequently more advanced and invasive in IPMNs. Silencing of the tumour suppressor genes p53, CDKN2A/p16/MTS1, STK11/LKB1, dual specificity phosphatase 6 (DUSP6), and Hypermethylation of numerous tumour suppressor genes, dual specificity phosphatase 6, deregulation of STK11/LKB1, silencing of the tumour suppressor genes p53, CDKN2A/p16/MTS1, STK11/LKB1, KRAS and protein kinase B/Akt pathways are among the oncogenic mechanisms that are activated in this process [53].

Cystic epithelial neoplasms that secrete mucin are known as mucin-producing cystic neoplasms. They make up roughly 2–5% of cancer tissues and are almost exclusively prevalent in women between 40 and 50 years. They also constitute 25% of pancreatic cysts that undergo resection [51].

## Conventional therapeutic strategies to treat pancreatic cancer

**Fig. 2:**
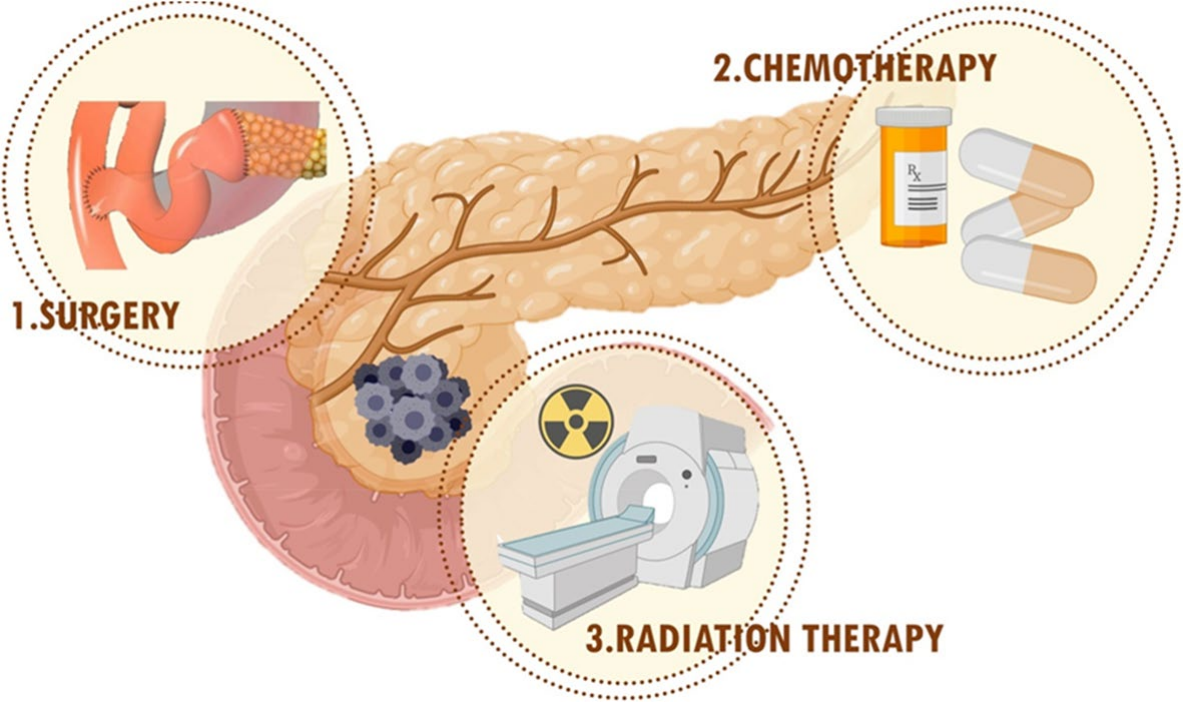
Conventional treatment approaches for pancreatic cancer

Surgery, chemotherapy and radiation therapy are the major conventional therapeutic choices for people with PC (Fig. 2), based on various factors and the cancer’s stage [54].

### Surgery

Surgery may potentially cure PC, and have shown to improve survival rates. The surgical treatments available are Whipple’s procedure, distal pancreatectomy, and total pancreatectomy. Surgery aims to achieve R0 resection and show good survival rates, which has been assisted by employing neo- adjuvant treatment and vascular resection to improve microscopic clearance. R0 resection refers to absence of any microscopic remains of the tumour in the primary tumour bed, while R1 resection is debated upon; Royal College of Pathologists describe it as a lesion inside 1 mm of the excision margin, whereas College of American Pathologists and Union for International Cancer Control describe it as microscopic indication of tumor cells at the determined resection boundary [50].

#### Biliary drainage vs. immediate surgery

PC patients often develop jaundice which can show complications such as coagulation disorder and postoperative infection. Hence obstructive jaundice is usually treated before surgery. There are more peri-operative problems when drainage is performed, according to a prospective multicenter randomised trial of endoscopic retrograde cholangiopancreatography (ERCP) with drainage vs. subsequent operation, which suggests that immediate surgery may show better results [50].

#### Anastomosis

The pancreatic remains can be anastomosed to the stomach or the jejunum after Whipple’s procedure. A major cause of morbidity is leak from the anastomosis. A recent meta-analysis has shown no reduction in formation of pancreatic fistula in either of the techniques, i.e., connecting the pancreas to the stomach mucosa or invagination into the jejunum [50].

#### Minimally invasive procedures

A first explained non-invasive pancreatic surgery was the laparoscopic distal pancreatectomy, which was revealed to have equivalent mortality and morbidity to open distal pancreatectomy, in addition to fewer loss of blood and a shorter hospital stay. However due to lack of level one evidence it cannot be classified as superior compared to open distal pancreatectomy. Another alternative is robotic surgery to improve Whipple’s procedure, but these require higher capital. A meta-analysis of retrospective cohort research suggests that the robotic group had a relatively low risk of side effects and less margin interference, but the absence of randomised in such experiments suggests that they could have selection bias [50]. ERCP is a well-established endoscopic process used to identify intermediate biliary systems and to perform presurgical biliary drainage in patients with pancreatic head cancer [55].

#### Arterial and venous resection

Vascular resection depends on the correlation between the pancreatic cancerous cells and the neighbouring vascular system. Feasibility vs. the benefit of resection must be carefully evaluated, as the meta-analysis of studies involving arterial resection showed poor outcomes for 1 year and three-year survival rates and higher rates of peri-operative mortality. Therefore, it is widely accepted that invasion of the superior mesenteric artery and coeliac trunk is a problematic to resection. However venous resection can show high potential results; studies were conducted on two groups of patients, one with and one without undergoing venous resection. Twenty-two retrospective cohort studies were meta-analysed and no difference was found in the peri-operative morbidity, one, or three-year survival in either group, but more blood loss and increased operative time were recorded in the former group. There is a possibility of selection bias due to lack of randomization [50].

### Chemotherapy

Gemcitabine was approved in 1996 by the FDA to be a first line drug in locally advanced or metastatic patients. There are numerous additional chemotherapeutic drugs, including 5-Fluorouracil and its derived products. Capecitabine is a 5-FU prodrug that is taken orally, S-1 is another oral derivative, which has been observed to be a single agent chemotherapeutic agent in a phase II trial which was well tolerated and efficient in individuals with metastatic tumour cells with a one-year survival rate of 15.8%. FOLFIRINOX is a novel and potent chemotherapy which combines oxaliplatin, fluorouracil, and leucovorin. Its phase II trial was promising as the median overall survival was 10.2 months. The phase III trial results aligned with the phase II results. Other drugs such as cisplatin and oxaliplatin are known which are not used in monotherapy but usually seen in combination therapy. Taxanes have been studied as chemotherapeutic agents, but paclitaxel has been observed to have minimal efficacy. However, paclitaxel and docetaxel are used for chemotherapy in patients who showed gemcitabine failure with good results. Irinotecan was tested as a monotherapy in a phase II trial where it showed mild toxicity and median survival duration was 5.2 months [56]. Current chemotherapeutic agents primarily inhibit tumour prognosis by inducing apoptosis. However PC cells, develop resistance to apoptosis via various mechanisms and hence combination approaches are favoured [57–61].

### Radiation therapy

The ionizing radiation of the high energy X-rays employed in radiotherapy cause damage to the cancer cell DNA, leading to apoptosis or cell cycle arrest. Ferroptosis, necroptosis, or pyroptosis may be seen in solid cancers as a result of radiotherapy. Radiation induced cell death is thought to be controlled by sensors (such as STING1), transcription factors (such as TP53), and DNA damage response related kinases (such as ATM). In an attempt to revive their tumorigenic potential, the dying cancer cells release HMGB1, which binds to the cancer stem cells via the TLR2 receptor. Identification of more DAMPs and their receptors is required to find out how tumour microenvironment is related to radiography [62].

### Palliative management

Palliative treatment is opted when the cancer becomes metastatic or irresectable. It involves symptom control, jaundice management, palliative chemotherapy with the preferred regimen of FOLFIRONOX (mFOLFIRONOX with 5-fluorouracil). A multicentre randomised trial was performed by Conroy et al. in 48 French centres where patients were given either gemcitabine or FOLFIRINOX. FOLFIRINOX showed higher median overall survival rate than gemcitabine, despite incidences of adverse effects. Since the PRODIGE trials, For people with metastatic PDAC, FOLFIRINOX is now considered the standard of care [50, 54, 63].

## Challenges associated with conventional therapy for treating pancreatic cancer in clinical settings

**Fig. 3:**
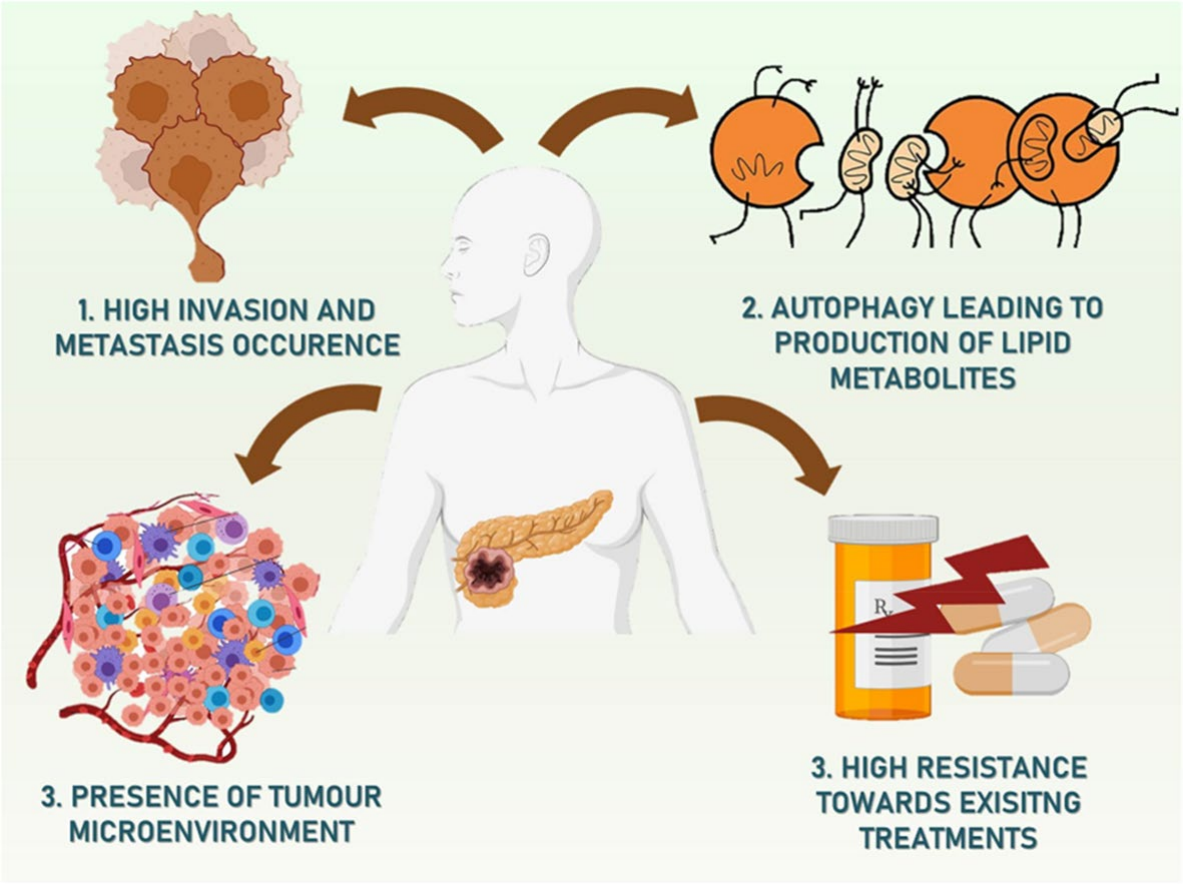
Hurdles in the path of treatment of pancreatic cancer

There are no viable treatments for the lethal disease PC. The inter and intra-tumour heterogeneity is partly to blame for this [64]. Identification of the molecular subtypes of PC may enhance clinical results by enabling the design of customized therapies [65]. Low tumour cellularity continues to be an important challenge in distinguishing the molecular subtypes of PC because of the desmoplastic character of this disease [66]. Numerous biological activities linked with tumour cells, such as cell differentiation, proliferation, immunological control, metabolism, angiogenesis, migration, and apoptosis, have been connected to signalling pathways (Fig. 3) [67]. Full knowledge of these pathways is probably going to be helpful in the creation of molecularly targeted PC treatments because they have been linked to the growth of tumours, prognosis, and treatment resistance in PC. Recent research has shown that transcription factors, growth factors, miRNAs, and cytokines are only a few of the substances that might cause an epithelial-mesenchymal transition in pancreatic cancerous cells [68]. EMT in pancreatic tumour cells has been shown to enhanced resistance of drug as well as a cancer stem cell phenotype [69].

### High invasion and tumour growth of pancreatic cancer

The microenvironment surrounding the pancreatic tumour is critical to the growth of PC. The microenvironment of the pancreatic tumour is altered through mutual communication between stromal and cancer cells, which can prepare the main tumour for cell migration and metastasis. Soluble substances and exosomes secreted by primary PC tissues could influence the localized microenvironment in tumour sites to enhance colonization [70]. Through the release of CXCL8, CXCL1, and VEGF certain stromal cells, such as TAMs, also take a role in the metastasis and angiogenesis of pancreatic tumour tissues [71].

### Reprogrammed lipid metabolism in pancreatic cancer

To maintain energy storage, and cellular structures, and produce signalling molecules, the cellular process of lipid metabolism transforms nutrients into metabolites intermediates. More evidence is pointing to the development of several malignancies, such as PC, as being correlated with disruption of lipid metabolism [72]. Cells can break down and reuse they possess cell structures through the cellular “self-eating” process termed autophagy, which improves their ability to survive in stressful situations. Importantly, it appears that autophagy plays contradictory roles in the development of cancer, among both tumour-promoting and tumour-suppressive actions that are probably context-dependent. Autophagy has a complex role in PC. Due to its role in maintaining cellular quality, autophagy is ant tumorigenic in the early stages of cancer. However, in more advanced cancers, autophagy would promote the survival rate of cancerous cells and their growth by supplying resources for biomolecule production and biogenesis [73]. Autophagy appears to be essential for pancreatic tumour cell metabolism and survival, according to a growing body of research [74].

### Tumour microenvironment of pancreatic cancer

A dense and extensive fibrous stroma, which can make up as much as 90% of the overall tumour volume, is a hallmark of PC. The stromal milieu and tumour cells interact in a complicated way, and stromal components govern the development of PC in an even more intricate way [75]. Hypoxic conditions can be produced by pancreatic tumours, which bring MDSCs to the tumour microenvironment in which they produce an immunosuppressive effect [76]. Conventional treatment strategies that concentrate on stromal desmoplasia depletion typically produce dismal outcomes. Future therapy methods that focus on the tumour and the stroma might prove more effective given the complexity of the PDAC microenvironment. In pancreatic tumour cells, CAF-derived EVs can boost glycolysis and Gln-dependent reductive carboxylation while decreasing mitochondrial OXPHOS metabolism. Gemcitabine treatment for PC may lead to an increase in the release of EVs produced from CAF, thereby increasing the lifespan of tumour cells and chemo resistance. Fibroblast activation protein is highly expressed in CAFs, which has been identified as a crucial factor accelerating PDAC progression [77].

### Conventional treatments

Only a small percentage of PDA patients have tumours that can be surgically removed, making total surgical excision the only option for long-term treatment. This is because tumours, by the time they are diagnosed, have typically progressed to include important abdominal arteries as well as other organs. Despite reductions in short-term morbidity and mortality at large facilities, significant technical advancements in surgical resection have been made. Even at the most skilled hospitals, post-surgery survival is dismal [78]. The majority of PC patients get therapies like radiotherapy and/or chemotherapy in addition to or after surgery, although due to certain therapies’ inherent resistance to apoptosis, their effects on survival rates are negligible. Therefore, it is urgently necessary to identify new molecular targets implicated in the development of PC as well as novel indicators for rapid recognition. Radiation is another potential strategy that can marginally increase the quality of life and patient survival, in addition to basic chemotherapy. Inhibiting mutant genes like KRAS and molecules involved in signalling pathways that significantly accelerate the development of the disease appears to be the most effective approach for treating PDAC. However, the majority of gene inhibition techniques are still only accessible in preclinical settings. Targeted drugs used in combination with chemotherapy have indeed been thoroughly researched. However, more critically, current research has shown that the key to successfully treating PDAC is the effective transport of therapeutic drugs into the tumour and tumour microenvironment, underscoring the importance of nanomaterials and nano delivery [36, 79–85]. The FDA has approved several agents and combinations of drugs for the treatment of PC, although folfirinox, gemcitabine, albumin-bound paclitaxel, and 5-fluorouracil are the most frequently prescribed. Due to its half-life being short and quick bodily clearance, gemcitabine is typically given in greater and repeated doses, which have many undesirable side effects [86]. Additionally, it has been explained that quickly develop resistance in tumour cells through variety of unexplained methods. Although clinical trials have shown that gemcitabine therapy improves survival, only 5–10% of PDAC sufferers respond to it. Erlotinib and gemcitabine together only slightly increase one-year survival rates by 23%. When compared to gemcitabine, which showed an overall survival of 6.8 months in people with metastatic PC, FOLFIRINOX considerably increases the survival rate by nearly 4 months [87]. The fact that PC is indeed challenging to cure with standard therapy is due to many reasons. PDAC exhibits a high occurrence of genomic alterations that cause significant genomic instability and may reduce the efficacy of therapy, particularly of targeted medicines, by causing subsequent or inherited chemo resistance. Disruption of suppressor activities in some genes, such as p53, can later cause increased genetic instability, a reduction in apoptosis, and diminished responsiveness to treatments. It has been demonstrated that the number of gene mutations and patients’ overall survival is strongly associated. PDAC patient is responding poorly to conventional therapies because of these coupled mutations. It is more challenging to treat because of the incredibly intricate system of genetic and signalling changes, and interactions between cells and their surroundings. In 12 separate signalling pathways that are often improperly active in most PC cases, there are about 63 genetic variations defined. The transport of chemotherapeutic drugs is likely to be hindered by hypo vascularity and strong desmoplastic response, which decreases the vulnerability of pancreatic tumour cells to insufficient dosages [88]. Due to the genetic heterogeneity of PC, conventional agents that focus on various cellular functions fail to discriminate between malignant and healthy cells, causing undesirable side effects. Therefore, the development of targeted therapeutics utilizing small-molecule inhibitors such as siRNA and aptamers that specifically target growth factors, cancer cell surface receptors, or other proteins involved in the overall evolution of the illness is required. Combination or targeted therapies are more effective than conventional treatments because they block an important signalling pathway that is a key regulator of cell progression, metastasis, survival, and proliferation [89].

## A cocktail of aptamer/siRNA mediated chemotherapy against pancreatic cancer

**Fig. 4:**
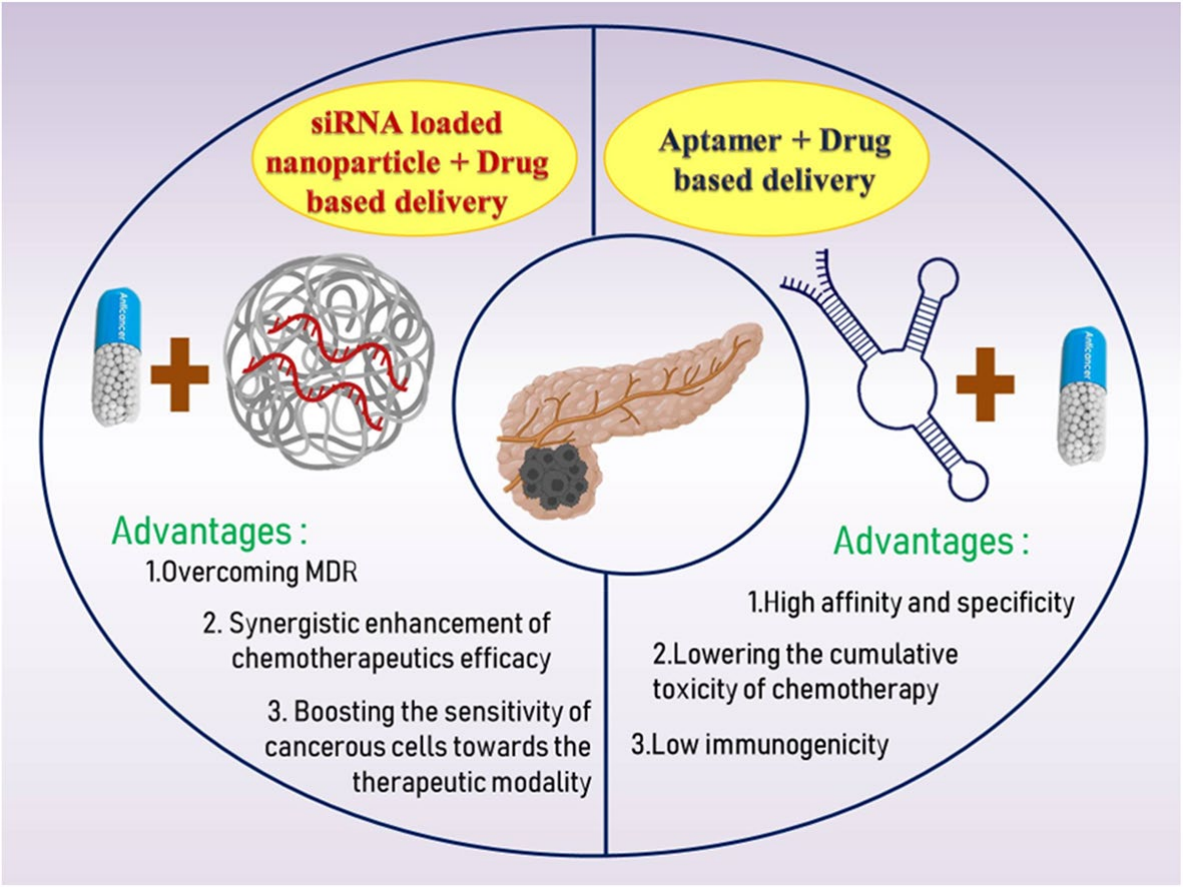
The combinatorial approach using siRNA and aptamer for pancreatic cancer and potential benefits

A deadly condition with a poor overall survival rate is PC. The majority of patients will undergo several types of multimodal therapy for PC. Surgery is the sole and most effective curative procedure. According to the American Joint Committee on Cancer’s staging method, which is currently in its eighth edition, it is followed by radiation therapy, adjuvant chemotherapy, or palliative care, based on their cancer’s stage. This staging method considers the TNM status, which stands for T - tumour size; N - lymph node involvement; and M - distant metastasis [90]. The most frequently prescribed therapy for people with advanced PC is chemotherapy. Systemic chemotherapy aims to reduce symptoms and increase survival time. The best therapeutic option for advanced PC is gemcitabine, which improves overall survivability just slightly. Unfortunately, people who have advanced PC only have an overall survival of between 5 and 6 months even with gemcitabine treatment, and only 5.4% of them respond to the drug. Nab-paclitaxel with modified folfirinox and gemcitabine are usually regarded as the ideal adjuvant chemotherapy treatments, even though they offer only limited survival advantages and high toxicity. Additionally, several novel targeted treatments have not been able to significantly improve survival rates, but erlotinib with gemcitabine had a marginally positive clinical effect. As a result, there is a significant need for new treatments, such as combination therapies and creative immunotherapies with stronger antitumor effects [91]. The overall survival increased with different combinations of gemcitabine-based therapies with either molecularly targeted or cytotoxic drugs. Multiple meta-analyses of randomized controlled trials have demonstrated that in people with advanced pancreatic adenocarcinoma, the gemcitabine with aptamer and siRNA results in a modest improvement in overall survival [63]. The 5-fluorouracil compounds S-1 and capecitabine have few therapeutic advantages. Patients suffering from pancreatic adenocarcinoma who have strong overall effectiveness may benefit from the novel and aggressive treatment represented by folfirinox. Other chemotherapy agents including irinotecan and platinum don’t significantly increase survival rates even though have been utilized in combination therapies. Regional intra-arterial chemotherapy, when compared to systemically administered chemotherapy, generates a better-localized concentration of drug in cancer cells with reduced systemic drug toxicity, and maybe a more effective treatment plan. Despite advancements in chemotherapy treatments for pancreatic adenocarcinoma, worldwide survival rates haven’t changed all that much in the past 10 years. The latest advancements in chemotherapy with molecularly targeted agents such as aptamers, siRNA, and nanocarrier have considerable potential to treat PC, particularly in cases where the illness has spread to other organs (Fig. 4). A significant body of preclinical and clinical research suggests that combinational therapies to a particular molecular targeted therapy improve the efficiency of the monotherapy despite enhancing toxicity [30–33, 92]. These therapies are viewed as a novel hope for the therapy of pancreatic cancer since it is thought to have synergistic benefits due to the diverse pharmacological effects it has on tumour cells [56].

### Combinatorial approach using aptamer and chemotherapeutics

Aptamers are peptide molecules or oligosaccharides, like single-stranded DNA and RNA, because of their distinct 3D structures, they attach to their targets with high affinity and specificity [39–42]. The folding and sequence patterns of ssDNA and RNA aptamers in particular might vary even though they interact with almost the same receptor. Scientists are paying close attention to aptamers since they not just possess many of the benefits of antibodies but also possess special qualities including limitless uses, low cost, and heat stability. Due to stability and easy modifications of aptamers, a wide range of analytical techniques, including mass-sensitive, optical, colorimetric, and electrochemical approaches, can be used to recognize the targets [93]. Because aptamers are tiny, they are simple to modify and can be connected to many nanoparticle processes to improve multiple functions. They also have a quick turnaround time, improved thermal stability, no batch-to-batch variability, cheaper manufacturing costs, and complete chemical synthesis. When tested for CEA measurement, aptamer-based biosensors displayed good affinity, biocompatibility, outstanding stability, and selectivity. An important mediator of inflammation known as interleukin-6 has been linked to PC patients as a predictive indicator or diagnostic biomarker [94]. The distinctive advantages of aptamers, such as their quick tissue accumulation abilities, ease of cell internalization, high binding affinity, and selectivity have caused aptamer-drug conjugates to develop into the most alluring methods for targeted therapy. Aptamer-drug conjugates are a novel targeted drug delivery mechanism that has been developed as a result of the successful inclusion of small drug molecules into aptamers by a covalent bond-forming technology. This innovation makes it possible to deliver potential anticancer cargoes selectively to specific tumour cells, lowering the cumulative toxicity of chemotherapy and increasing its effectiveness. Due to the numerous benefits of these aptamers, which include deep tissue penetration, limited immunogenicity, site-specific modifications, easy production, high affinity, and impressive specificity, the aptamer drug conjugates developed by the conjugation technique were the subject of extensive research in the past few years [95]. As aptamers are created using a chemical technique, the development of ApDCs is simpler and more practical than that of antibody-drug conjugates [96]. Treatment for pancreatic carcinoma cells with aptamers can be divided into three groups: (a) termed target inhibitors, in which the aptamers attach to the proteins associated with tumours and prevent the proteins & their downstream processes from functioning; (b) as chemotherapeutic agents, in which aptamers transport the therapeutics to the target sites; (c) efficient genetic delivery, in which the target gene is carried with gene regulatory components to control tumour [97]. The following section highlights numerous strategies specifically used for aptamer- chemotherapeutic agents’ combination in PC (Table 1).

**Table 1:** Application of aptamer and chemotherapeutics combination strategies in the therapy of pancreatic cancer

S.No.	Aptamer	Drug	Targeting ligand	In vivo model	Route of administration	Cell line	Result	Ref.
1.	Wnt5a and FZD7	Gemcitabine	ABCG2	–	–	Capan-2	Wnt5a-induced resistance of gemcitabine in pancreatic tumour cells.	[98]
2.	APTA-12	Gemcitabine	Nucleolin	Mouse	Intravenous	Capan-1	Considerably increased targeting affinity and suppression of cancer cell growth.	[96]
3.	P12FR2	2′-fluoropyrimidine	PAUF	Mouse	Intraperitoneal	CFPAC-1	Inhibit metastasis and cell proliferation	[99]
4.	E07	Gemcitabine	EGFR	–	–	MiaPaCa-2 and HPAF-2	Targeted therapy increases effectiveness and reduce side effects.	[100]
5.	AS1411	AuNS	Nucleolin	–	–	PANC-1	Increase cell death and caspase activity in tumour cells.	[101]
6.	AP1153	CPSNPs	CCKBR	Mouse	Intraperitoneal	PANC-1	Enhanced delivery and absorption to pancreatic tumour cells.	[102]
7.	Waz and E07	MMAE and MMAF	EGFR, TfR	–	–	Panc-1, MIA PaCa-2, and BxPC3	Delivery of hazardous payloads specifically to pancreatic tumour cells.	[103]
8.	Ap52	Doxorubicin	MAGE-A3	Mouse	Intravenous, In situ	AsPC-1	Provide targeted drug delivery and combined modulation of cell immunity for cancer treatment.	[104]
9.	NF-κB	Doxorubicin	TfR	–	–	MIA PaCa-2	Improving the Dox-mediated apoptotic impact in the pancreatic cancer cells.	[105]
10.	P19	Gemcitabine, 5-FU and MMAE	–	–	–	AsPC-1	Significantly reduced cell growth and produced mitotic G2/M phase arrest.	[106]

Zhang and colleagues developed a unique treatment approach for PDAC by elucidating the molecular processes underpinning Gem resistance. The ATP-binding cassette transporter superfamily proteins are considered to have significant aspects in MDR. And it has been demonstrated that PC cells need an increase in ABCG2 protein expression to be resistant to Gem. This conclusion is similar to the current findings, which showed that three distinct Gem-resistant pancreatic tumour cells had considerably higher protein expression of ABCG2 as compared to parental control cells. In addition, IHC staining of 24 PDAC and neighbouring tissue samples showed that poor prognosis was associated with increased ABCG2 expression. It would be advantageous to investigate if the poor prognosis brought on by the more expression of ABCG2 was related to an increase in vivo drug resistance. In conclusion, the expression of ABCG2 and the resistance of PC cells to anticancer drugs are both controlled by proteins in the Wnt pathway, such as Wnt5a and FZD7 [98]. Park and colleagues produced an ApDC called AS1411, APTA-12, which contains gemcitabine, and showed that the combination has significant chemotherapy efficacy for PC. The study aimed to use oligonucleotide aptamers to deliver the chemotherapeutic drug gemcitabine; hence the incorporation of the aptamer AS1411 was selected as a model for this study. The internal stem-loop structure of AS1411, which is located within 12 and 15 nucleotides, is said to produce a stable G-quadruplex structure. The relative stability of G-quadruplex-forming oligonucleotides over nuclease degradation is well established. The robust G-quadruplex configuration of guanine-rich APTA-12 explains its remarkable serum stability. For Capan-1 cells, it was discovered that APTA-12 has a substantially stronger affinity. These findings imply that nucleolin was the only protein to which the aptamer APTA-12 bound. W3As compared to Capan-1 cells treated with AS1411, APTA-12-treated Capan-1 cells had IC50 values that were 43-fold lower. An in vivo analysis revealed that the APTA-12 therapy had no significant adverse effects or toxicity. These findings imply that targeted pancreatic treatment of cancer using aptamer-based gemcitabine transport of APTA-12 is a viable anticancer approach [96].

In a study by Kim and his associates, an RNA aptamer (P12FR2) modified with a 2′ fluoropyrimidine targeted at human pancreatic adenocarcinoma up-regulated factor was characterized and generated. Pancreatic tumour cell’s proliferation and metastasis are mediated by PAUF, which is significantly abundant in pancreatic adenocarcinoma. P12FR2 has an approximated apparent K (D) of 77 nm and binds exclusively to human PAUF. In a wound healing study, PANC-1, human pancreatic tumour cell line, is inhibited by the P12FR2 aptamer from migrating in response to PAUF. Additionally, intraperitoneal injection of P12FR2 prevented weight loss in the treated animals while reducing tumour progression in an in vivo xenograft model employing CFPAC-1 PC cells by roughly 60%. In conclusion, we suggest that P12FR2, a PAUF-specific RNA aptamer, has the promising strategy to be useful in the treatment of pancreatic adenocarcinoma [99]. Ray and co-workers developed an RNA aptamer that transfers polymers containing the drug gemcitabine to the EGFR-dominant PC cells and prevents the growth of those cells. The RNA aptamer is resistant to nuclease enzyme and is incorporated by epidermal growth factor receptor. Cellular metabolism and absorption, as well as reduced tumour perfusion, have all been linked to Gem resistance. Some of these chemo resistance pathways might be circumvented by the suggested Gem delivery method. The EGFR aptamer (E07) has a significantly larger extent to attach to the MiaPaCa-2 cell line because it has high EGFR-expression cells than the HPAF-2 cell line because of the low EGFR-expressing PC cell and it is determined by using microscopy and flow cytometry. A novel strategy for treating pancreatic adenocarcinoma is the use of aptamers for cell-type-specific delivery. This strategy can be more broadly applicable to certain another cell surface receptors and various kinds of therapeutic cargo, such as photosensitizing agents, radioisotopes, siRNAs, toxins, and other chemotherapeutic drugs. By lowering the overall dose of gemcitabine needed and reducing absorption into healthy cells, this method has the promising capability to reduce hazardous side effects of drugs [100]. Dam and his co-worker developed a nanoconstruct that can target the prevalent protein nucleolin and act as a cell-type independent agent. In a group of 12 carcinoma lines having four distinct subgroups, gold nanostars (AuNS) coated to high levels of nucleolin-specific DNA aptamer AS1411 (Apt-AuNS) exhibited antitumor activity. When compared to cells devoid of the nanoconstructs, Apt-AuNS caused a 200% reduction in the expression of the ant apoptotic Bcl-2 mRNA. In comparison to tumour cells treated with unbound AS1411 at more than 10 times the concentration, Apt-AuNS treatment boosted the cell death and apoptosis 3/7 activity by 17% and 1.5 times respectively. The in vitro effectiveness of the nanoconstructs in the carcinoma line group was further improved by light-triggered expulsion of aptamer from the AuNS, which resulted in a 40% reduction in cell survival and a 2-fold rise in upregulation when compared to therapy with Apt-AuNS alone. Importantly, the aptamer-drug durability was raised and comparatively high concentrations of AS1411 were presented as a result of the packing of AS1411 onto AuNS nanocarriers, which considerably increased the in vitro efficacy. We predict that Apt-AuNS can serve as an opportunity for a new class of cell-type independent medicines that might overcome some present issues in targeted therapy because it exhibits antitumor activity on the PC cell line that is regulated by the widespread protein nucleolin [101]. Clawson and colleagues developed DNA aptamers (APs) that attach to the G-protein-coupled cholecystokinin B receptor and also their characteristics and effectiveness as targeted therapy. An important step in achieving that goal is the characterization and identification of DNA APs having a strong affinity for the CCKBR, a cell membrane protein that is present in all human pancreatic adenocarcinoma cells. APs were chosen to bind to unique locations on the extracellular N-terminal of the cholecystokinin B receptor and can target calcium phosphosilicate nanoparticles to pancreatic tumour cells without triggering anti-apoptotic and pro-proliferative receptor signalling. These aptamers could aid in the rapid recognition of pancreatic tumours because researchers showed that CCKBRs are indeed detectable on antecedent PanIN tumours. The capacity to comprehend precursor tumours prior to they develop into full-blown PC and disseminate should better patient outcomes because therapy could be curable if tumours are found early. The interface of luminescent nanoparticles was substantially facilitated by AP1153 bio conjugation and nanoparticles were administered to pancreatic tumours in vivo. This aptamer-targeted nanoparticle delivery platform’s selectivity offers a promising strategy for both improved chemotherapeutic care for PC patients and improved early diagnosis of pancreatic adenocarcinoma lesions [102]. Kratschmer and Levy created simple and reliable aptamer-auristatin combination, and they are poisonous to pancreatic adenocarcinoma cells. On three PC cell lines, researchers tested the anti-EGFR aptamer E07 and anti-transferrin aptamer Waz conjugated to MMAF and MMAE. Results indicate that binding affinity alone cannot be used to choose the optimum aptamer for delivering a toxin to a specific cell line. All three cell lines showed low IC50 values for aptamer-monomethyl auristatin E combination, although C36-MMAE conjugates also showed low IC50 values, indicating a very small therapeutic window. For MMAF conjugates, the broadest therapeutic window was discovered. Future research ought to investigate other linker and toxin combinations as well as how to increase aptamer persistence for in vivo application. This observations indicate that ApDCs combination are a feasible option with numerous potential benefits, despite the fact that ApDCs have received a lot of attention in the literature for the treatment of pancreatic adenocarcinoma [103]. Wang and his associates demonstrated that Ap52, a DNA aptamer against the specific cancer protein MAGE-A3111–125, efficiently penetrates the cancer cell in a pancreatic xenograft mouse model and preferentially transports Dox to many cancer cell types, including skin, pancreatic, oral, and breast cancer cells, leading to cytotoxicity. As a result, Ap52 can be identified as a potential agent for the detection and treatment of cancer. This distinct category of the aptamer-drug complex may offer drug targeting and combinatorial manipulation of cell immunity for the treatment of cancer, in addition to the development of tools for tumour diagnostics. To combat tumour heterogeneity, aptamers that address a variety of common tumour-specific antigens could be utilized in tandem. The in vivo studies of AsPC-1-implanted mice were studied, and the results show that phosphorothioate-modified Ap52 concentrates in the tumour of xenograft after in situ or intravenous injection. ThioAp52 can deliver Dox anticancer medication to different types of tumour cells, such as those in the pancreas, breast, mouth, and skin. According to image analysis, pure Dox is absorbed by all cell lines, whereas the ThioAp52-Dox combination permeates tumour cells. A combination of ThioAp52 and Dox has more cytotoxicity against cancer cells than against equivalent healthy cells. These findings suggest that this aptamer against a specific malignant cell antigen may have a significant promising strategy as a therapeutic delivery system for different tumours [104]. Porciani and his associates introduced a molecular engineering method to create a unique oligonucleotide combination drug delivery platform made of a DNA decoy and an RNA aptamer. Using an RNA aptamer against the ant transferrin receptor, this assembly can focus cancer cells and deliver doxorubicin and an NF-κB decoy ODN, which disables a cell-survival protein, in a targeted manner. The self-assembled complex of DNA was formed between 2 complementary ssDNA sequences, one covalently joined to nuclear factor- κB and the other embedded in the targeted RNA aptamer by using a disulphide bond. In the conditions in which a reductive environment and low pH are present in endolysosomal compartments, both payloads are discharged. Western blot analysis, cell viability and confocal microscopy were used to evaluate the oligonucleotide chimera’s cytotoxicity and targeting. These findings show that the NF- κB decoy indeed decreases NF-κB activity, which eventually increases the antitumor efficiency of doxorubicin specifically in cancerous cells [105]. .Yoon et al. describe the combination of P19 RNA aptamer and cytotoxic drugs to enhance the therapeutic index of chemotherapeutic agents and is used for the treatment of pancreatic adenocarcinoma. P19 was conjugated to Derivative of Maytansine 1 (DM1) and MonoMethyl Auristatin E (MMAE), or it was combined with 5-Fluorouracil and gemcitabine. The ApDCs dramatically reduced cellular progression by 54–34% in the gemcitabine-resistant AsPC-1 PC cell line and by 51–53% in the PANC-1 cell line by activating the phosphorylation of histone H2AX on Ser139. Compared to the control group, P19-DM1 and P19-MMAE produced mitotic G2/M phase arrest and decreased cell growth by as much as 56%. These findings imply that this strategy could help reduce cytotoxic side effects in healthy tissue. At 72 hours, AsPC-1 and PANC-1 cells were considerably less proliferative when treated with P19-5FdUMP and P19-5FdUMP. In this situation, a researcher showed that ApDCs can specifically internalize tumour cells with an extreme metastatic phenotype, administer efficacious cytotoxins and anti-metabolites through direct delivery, and induce robust proliferative action while protecting healthy cells. From this investigation, we were able to determine that ApDCs integrated into cells and caused DNA damage. This study implies that since limited penetrance of the cell is the most plausible model for gemcitabine resistance, ApDCs may aid in the elimination of chemo resistance in PC by enabling administration and transport of the medication to unreachable cells. This finding suggests that the P19 aptamers could serve as a potential for antitumor drug delivery to pancreatic cells [106].

### Combinatorial approach using siRNA and chemotherapeutics

Cancers are exceedingly heterogeneous in nature and are prone to show resistance to the therapy provided. Cancerous cells develop resistance to therapies such as chemotherapy, photodynamic therapy and radiation therapy [107]. Several bio-molecular processes like increased drug efflux and overexpression of anti-apoptotic genes have been identified depicting the mechanism of resistance however it is considered complex and beyond the capacity of the human brain [108, 109]. The emergence of multi-drug resistance (MDR) is a major concern for cancer treatment. It eventually leads to a decrease in intracellular concentration of the drug, limiting apoptosis and thus subsequent cytotoxicity. RNAi has the potential for down-regulation of specific genes and thus has evolved into a viable tool for treating PC as the stromal barrier and growth of the tumour is attributed to gene mutation and disrupted signalling for the conventional therapy [110]. siRNA is considered a missile in RNAi technology that can silently inhibit the expression of specific genes involved in the MDR. The combinatorial approach of a chemotherapeutic agent with siRNA offers several benefits by overcoming MDR and synergistically enhancing chemotherapeutics efficacy or boosting the sensitivity of cancerous cells towards the therapeutic modality [49]. However, the potential of combinatorial delivery of siRNA with chemotherapeutic agents is met with several challenges limiting the efficacy such as inadequate tumour delivery, rapid clearance and serum degradation [111]. Furthermore, PC’s rich fibrotic stroma, as well as vascular barriers, complicates the delivery of siRNA to the tumour and adjacent cells [112]. Hence to get the required therapeutic action from the combinatorial approach efficient nanoparticle-mediated delivery is required. The following section highlights numerous methods of siRNA delivery using nanoparticle systems and other strategies specifically used for siRNA- chemotherapeutic agents’ combination in PC **(**Table 2**).**

**Table 2:** Application of siRNA and chemotherapeutics combination strategies in the therapy of pancreatic cancer

S.No.	Nanocarrier/ Other type of Carrier	Targeting ligand	In vivo model	Route of administration	Combination	Cell line	Result	Ref.
1.	Lipid NP	HIFIα	Mouse	Intravenous	siRNA and GEM	Panc-1	Prevention in burst release and boosted cytoxicity of GEM	[113]
2.	Lipid NP	HIFIα	Mouse	Intravenous	siRNA and GEM	Panc-1	Increased intracellular concentration and reduction in tumour weight	[114]
3.	Lipid NP	KRAS	Mouse	Intravenous	siRNA and GEM	Panc-1	Decreased tumour proliferation, enhanced apoptosis and no toxicity	[115]
4.	Lipid NP	Mcl-1	Mouse	Intravenous	siRNA and GEM	BxPC3 and Panc-1	Higher cellular uptake, prevention of siRNA from nuclease degradation and overcomes GEM resistance	[116]
5.	Lipid NP	RRM2	Mouse	Intraperitoneal and Peritumoral	siRNA and DOX	Panc-1	Reduction in tumour growth, decreased metastasis and DOX dose	[117]
6.	Polymer NP	PLK 1	Mouse	Intraperitoneal	siRNA and GEM	KPC8060 and S2–013	Greater accumulation in tumours and significant increase in anti-cancer activity	[118]
7.	Dendrimer	ITCH	Mouse	Subcutaneous	siRNA and GEM	Mia-PaCa-2, BxPC3 and Panc-1	Significant gene knockdown and increased chemo sensitivity	[43]
8.	Gold NP	KRAS	Mouse	Intratumoral	siRNA and DOX	Panc-1	Reduction in tumour growth by 90%	[119]
9.	Magnetic NP	PD-L1	Mouse	Intravenous	siRNA and GEM	PAN-02	Supressed tumour growth and improved survival rates	[120]
10.	Implant	KRAS	Mouse and Human Patients	Subcutaneous	siRNA and GEM	–	Sustained release of drug, prevention of siRNA from serum degradation and enhanced overall survival rates	[121]
11.	Biodegradable construct	CHK1	–	–	siRNA and GEM	Mia-PaCa-2, BxPC3,Panc-1 and CEPAC-1	Reduction in GEM dose and enhanced efficacy	[122]
12.	Polyester based vectors	Notch-1 and KRAS	–	–	siRNA and GEM	Mia-PaCa-2	Increased apoptosis and chemo sensitivity	[123]

#### Nanoparticle-assisted co-delivery of siRNA and chemotherapeutics

##### Lipid-based nanoparticles

Lipid-based nanoparticles or liposomes are used for a long time for transporting chemotherapeutic drugs and nucleic acids inside the body [124–127]. The efficacy of the encapsulated material is affected by its interactions with the carrier’s lipid components [128–131]. An ionic interaction occurs between negatively charged siRNA and the positively charged liposomal membrane of cationic lipids forming a siRNA-liposomal complex [132]. The stability of siRNA stability within the liposomes can be improved by condensing with polymers such as PEI or protamine [133]. Chemotherapeutic agents can be incorporated into the aqueous core or non-aqueous lipid bi-layer depending on their solubility. Various liposomal nanocarriers have developed for the co-delivery of siRNA and chemotherapeutic agents such as PEGylated liposomes and targeted liposomes [132].

Over the last decade, Hypoxia-inducible factor 1α [HIF1α] is identified as a potential target in treating PC. Overexpression of HIF1α promotes drug resistance to Gemcitabine (GEM), the current standard care for PC treatment. Zhao and co-workers developed a lipid-polymer hybrid nanoparticle (LENP) construct using PEGylated phospholipids and cationic e-polylysine-co-polymer (ENP) for co-delivering GEM and HIFIα-siRNA into Panc-1 human PDAC cells and xenograft mouse model. The ENP layer efficiently attracts the negatively charged HIFIα-siRNA onto its surface while incorporating the GEM in the aqueous core. The lipid bi-layer protects the nanoparticles to form aggregates, HIFIα-siRNA from serum degradation and burst release of GEM in the bloodstream. The co-delivery considerably boosted the cytotoxic and anti-cancer action of GEM. The formulated nano-complex showed higher stability and efficiently suppressed the expression of HIFIα both in vitro and in vivo. Furthermore, the LENP carrying GEM and HIFIα-siRNA demonstrated effective inhibition of tumour spread in the orthotopic tumour model and significantly reduced innate immunity activation by siRNA [113]. In another study Lin and associates formulated a GE-11 peptide linked liposomes incorporating GEM and siRNA against HIFIα. Conjugated peptide GE-11 enhanced the targeting ability of the liposomes and increased its concentration intracellular. The results depicted that the combinatorial delivery decreased the tumour weight by 4 times in comparison to the control group. Additionally, the tumour weight was decreased by 2-fold only when GEM was administered alone. siRNA efficiently reduced the HIFIα gene expression in cancerous cells. Overall, the liposomal preparation provides a unique platform for the co-delivery of HIFIα-siRNA and GEM for targeted PC treatment [114].

Mutation in the KRAS gene is frequently associated with human cancers, with 90% found in PC. Direct suppression of the mutant KRAS gene is quite challenging, despite its significant role in various malignancies across humans. Thus, to circumvent the obstacles, Wang and his co-workers created apolipoprotein-E3 (ApoE3) conjugated liposomes combining GEM and siRNA targeted against the KRAS gene. LDL receptors overexpressed on the surface of PC cells mutated with the KRAS gene, ApoE3 interacts with LDL receptors resulting in a significant increase in uptake of liposomes by the Panc-1 cells. The co-delivery of GEM and KRAS-siRNA synergistically boosted cell apoptosis and reduced cell viability in comparison to a single treatment. It was found that the anti-tumour effect of KRAS-siRNA was responsible for lowering the IC50 value. Suppression in the expression of the KRAS gene resulted in decreased proliferation in tumour cells, enhanced apoptotic effect, and suppressed tumour growth while showing no cytotoxicity. Thus, combinatorial delivery has a high potential for treating PC [115].

Myeloid cell leukaemia (Mcl-1) is an anti-apoptotic factor implicated in the cell death of various human cancers and has evolved as a possible target in PC therapy. Overexpression of Mcl-1 in PC cells diminishes their sensitivity toward GEM and is thus responsible for resistance toward GEM. Wang and his team created a lipid-based nanoparticle system (LPs) incorporating GEM combined with siRNA targeted against Mcl-1 (Mcl-1-siRNA) to boost GEM sensitivity and minimise its delivery challenges. The findings revealed that the combination delivery exhibited higher cellular uptake, down regulated Mcl-1 and substantial cytotoxicity. Furthermore, increased inhibition in tumour in vivo depicted that the LNP-GEM-Mcl-1-siRNA has better anti-tumour efficacy on comparing to individual administration of GEM or Mcl-1-siRNA alone, thus proving the synergistic effect of co-delivery. The histological evaluation revealed that LPs were able to co-deliver GEM and Mcl-1-siRNA at the same tumour site, hence overcoming GEM resistance and preventing siRNA serum degradation. Thus, the formulated LPs can be considered useful and efficient carriers for combinatorial delivery in treating PC [116]. In another study, Zheng and associates formulated a lipid nanoparticle (LNP) system loading Doxorubicin (DOX) and siRNA against RRM2 (siRMM2). The co-delivery of siRMM2 significantly increased the efficacy of DOX by 4 times in comparison to DOX alone, thus indicating synergism between siRMM2 and DOX. Furthermore, findings revealed that LNP-DOX-siRMM2 dramatically reduced tumour metastasis in the Panc-1 xenograft mouse model. The inhibitory activity was seen by the reduction in tumour weight by more than 40%. The quantitative analysis demonstrated a roughly 70% decrease in colonies. As a result, a high synergistic effect was observed and accomplished, as using siRNA in combination with DOX lowered the dose of DOX markedly and in turn, DOX also boosted the anti-tumour efficacy of siRNA. Thus this study provides a potential therapy for the treatment of PC [117].

##### Polymeric nanoparticles

Polymers provide a potential nanoparticle delivery system as they exhibit attractive characteristics like tunable size, surface modification and effective payload packing of the material inside it via ionic interactions [132]. Because of these characteristics, the entrapped material has more physical and chemical stability as well as exhibit consistent and controlled release pattern throughout use. They are considered ideal for carrying numerous agents inside them at the same time, such as siRNA and chemotherapeutics [14, 134–138]. Cationic polymers such as chitosan, PEI, and PLL are frequently used for gene delivery. Atelocollagen and PLGA which are amphiphilic have also shown potential for gene delivery applications [132].

Chemokine receptor – CXCR4 and polo-like kinase 1 (PLK1) play an essential role in metastasis and chemotherapeutics resistance in PC. To circumvent the hurdle for efficient treatment of PC, Tang and his co-workers developed a nanoparticle delivery system using cholesterol modified with co-block polymer PAMD encapsulating GEM and siRNA targeted against PLK1 (siPLK1). During their in vitro investigation, they discovered a remarkable synergism between polymeric CXCR4 antagonist PAMD-cholesterol and GEM in both humans’ pancreatic and murine cell lines. The nanoparticle’s bio distribution pattern revealed that they significantly accumulated inside primary and metastatic tumours. In vivo profiles of the nanoparticles demonstrated that the combined delivery of PAMD-CHOL-siPLK1-GEM showed increased anti-tumour activity in comparison to a single control. Thus it can be concluded that combinatorial delivery was proved to be beneficial in treating PC [118].

Dendrimers are tree-like, multi-branched, low-molecular-weight polymeric nano-carriers consisting of a core and inner and outer shells [139–146]. The inner shell is composed of numerous repeating layers of monomeric units (known as generations) created by chemical reactions, while the outer shell consists of several functional groups that determine the dendrimer’s properties [144, 146–151]. Polyamidoamine (PAMAM) dendrimers are among the most extensively researched dendrimers for gene delivery applications. PLL dendrimers and peptide dendrimers are other forms of dendrimers available [152–158]. ITCH E3 Ubiquitin ligase is an enzyme that is responsible for regulating the immune system in humans [159]. It is responsible for proteasome-dependent degradation of protein p73 which is important in regulating the cell cycle and apoptosis pathway. Fuente and his colleagues formulated a G3-PAMAM dendrimer (DAB-Am16) for co-delivery of GEM and siRNA against ITCH to Panc-1 and MIA PaCa-2 cell lines. The formed dendrimer complex revealed enhanced cellular uptake and greater inhibition of gene in vitro. The co-delivery of GEM showed a synergistic effect and remarkably increased gene knockdown in mice xenograft models on i.e., administration. The combination approach also increases the sensitivity of pancreatic cells toward chemotherapy. Hence, this approach is considered a promising therapy for PC [43].

##### Inorganic nanoparticles

Inorganic nanoparticles are efficient delivery systems for co-delivery of siRNA and chemotherapeutics as they exhibit distinct physicochemical characteristics including compact size, large surface area, biological stability, easily modifiable surface and magnetic nature [160]. Iron oxide, gold, mesoporous silica, carbon-based and magnetic nanoparticles are examples of commonly used inorganic nano-carriers for the combinatorial approach [154, 161–164].

Gold nanoparticles or nanorods (AuNPs) appear to be the best delivery systems as they are biocompatible, stable and the surfaces are easily functionalised [154, 164, 165]. siRNA forms electrostatic bonds with AuNPs or it can be conjugated with AuNPs by first absorbing it onto a layer of cationic polymer that is further deposited onto the surface of AuNPs. The polymer coating is often used for the co-delivery of siRNA and other therapeutics as it provides additional stability to the NPs [154, 161, 162, 164, 166, 167]. Yin and his co-workers formulated AuNPs with polyelectrolyte polymer coating incorporating KRAS-siRNA and Doxorubicin (DOX). The synthesised gold nano-complex suppressed tumour growth for approximately 25 days by controlled delivery of DOX and KRAS-siRNA in Panc-1 cells and subsequent exposing of the nanocomplex with 665 nm light. According to the researcher’s findings, the synergism of AuNPs with 665 nm light reduced the tumour growth by 90% in vivo. The superior anti-cancer effect is attributed to the inhibition of the KRAS gene resulting in S-phase arrest of the cell cycle in Panc-1 cells. Thus, the formulated nano-complex is a promising approach for the enhancement of therapeutic action in PC treatment [119].

Yoo and his colleagues prepared a magnetic nanoparticle (MN-siPDL1) incorporating GEM in combination with siRNA (siPDL1) targeted against a programmed death ligand (PD-L1). The formed nano-complex resulted in considerable tumour growth suppression and enhanced survival rates in administered mice. Two weeks after starting the therapy, a 90% decrease was observed in tumour volume. The treatment group showed no signs of mortality till week 5 and around 67% lived for 12 weeks whereas the control group showed a 100% mortality rate. Thus, this approach can be considered efficient for treatment and increase the 5-year survival rate [120] . The majority of inorganic nanoparticles have the potential for co-delivery of siRNA and chemotherapeutics in treating PC but they are still in their infancy stage as much literature is not available. They have to be yet evaluated for their material-related and in vivo toxicity. The lack of knowledge regarding their safety, efficacy and pharmacokinetic profile is seen as a key barrier impeding the use of inorganic nanoparticles for PC treatment.

### Other strategies for co-delivery of siRNA and chemotherapeutics

**Fig. 5:**
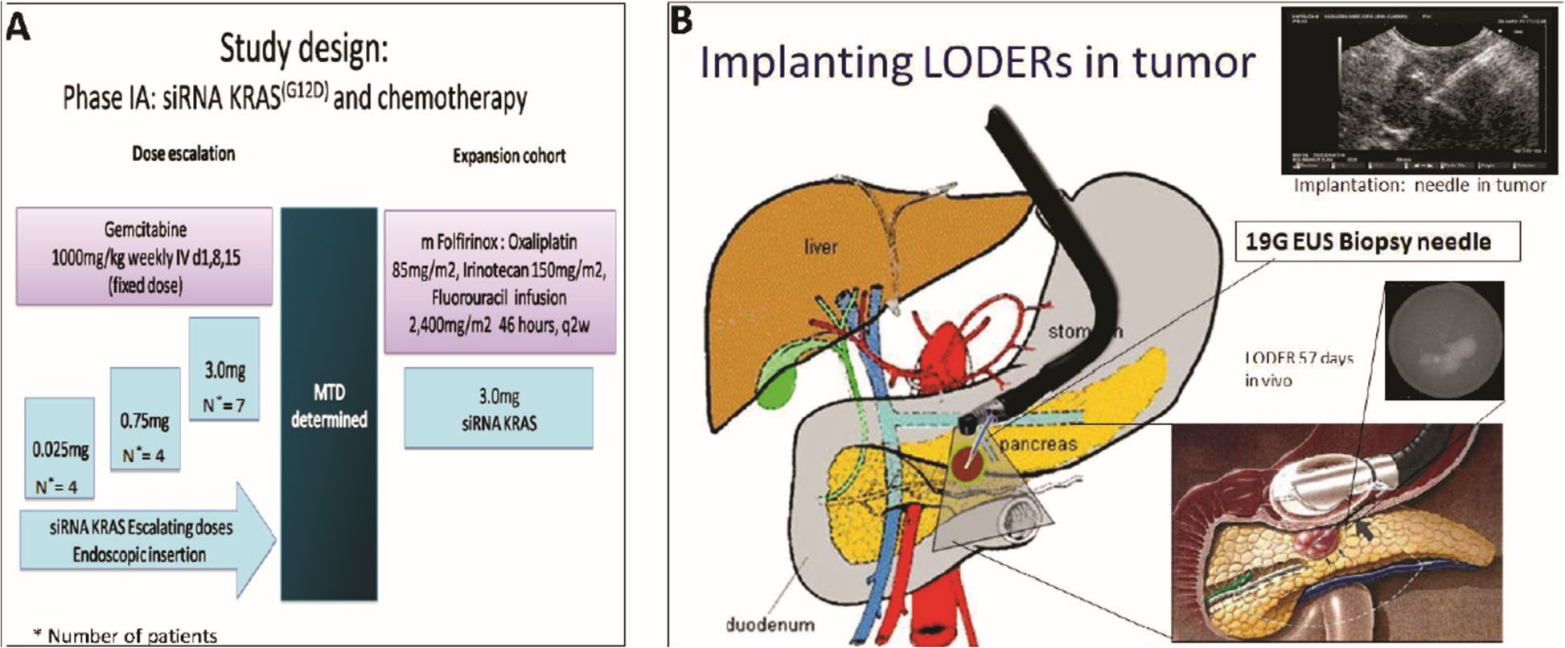
**A** Study design. **B ***siG12D-LODER™* is placed with Endoscopic US biopsy needle. Reproduced with permission from [121]

The accumulated genetic mutations trigger the activation of several types of oncogenes and silencing of tumour suppression genes. It is considered to be linked with epithelial carcinomas. Mutation in the KRAS gene has been recommended as a bio-marker for PC [168], as it is mutated in more than 90% of pancreatic ductal adenocarcinoma (PDAC) [88]. For treating patients with local advanced PC (LAPC), an efficient, biodegradable and non-toxic implant was created by Silenseed Ltd. named LODERTM (Local Drug EluteR). The implant consists of a biodegradable polymer matrix with anti-KRASG12D siRNA (siG12D) embedded inside it. It is intended to deliver the drug in a delayed and sustained manner inside the tumour over a period of several months, whilst protecting the siRNA from serum degradation [121]. Golan and his associates investigated the implant loaded with GEM and siG12D targeted toward LAPC. During the investigation they found that out of 15 enrolled, 12 did not show any signs of tumour progression and 2 showed a limited response, indicating an enhanced overall patient survival rate. 70% of patients showed a significant decrease in bio-marker CA19–9 for tumours. The relative survival rate was at 15.2 months and at 18 months was 38.5% only. Thus, this approach is considered, safe, tolerable and has the potential for treating patients with LAPC **(**Fig. 5**)** [121].

Gemcitabine is a non-nucleoside analogue and has been considered the standard chemotherapeutic agent for PC. The drug shows good efficacy in vitro but due to limited absorption, it must be administered in large amounts via infusion, which leads to substantial toxicity for the patient. Studies have found several genes that when suppressed by siRNA synergistically act with GEM and thus effectively reduce the dosage of GEM required for therapeutic action. However, it is worthy to note that the synergistic effect is only achieved if the GEM and siRNA enter the same cell simultaneously. Simonenko and his colleagues formulated a unique GEM-siRNA construct encapsulating GEM covalently linked to siRNA against targeted sites that are synergistic with GEM such as CHK1 and RAD17. They substituted a few specific base pairs in siRNA with GEM which led to an increase in efficacy. On comparing the GEM alone with the construct, the GEM-siRNA construct demonstrated 5–30 times increase in potency. Furthermore, combining the CHK1-GEM-siRNA construct with WEE1-siRNA construct culminated in 10-fold increase in IC50 value in comparison to GEM alone. Thus these constructs represent a potential therapeutic strategy to treat PC effectively [122].

The Notch1 gene is responsible for regulating cancer cell differentiation, proliferation and maintenance in many different kinds of cancers including PC [169, 170]. Yang and his co-workers created biodegradable charged polyester-based vectors (BCPVs) for co-delivery of Notch1-KRAS siRNA and GEM for administration into PC epithelial cell line Mia-PaCa-2. The codelivery of KRAS-Notch1 siRNA with GEM showed a synergetic effect leading to enhanced anti-tumour effect and apoptosis. Thus it is concluded that the mentioned combination therapy increases cellular death and promotes chemo sensitivity, leading to effective outcomes in patients [123].

## Future outlook

PC is one of the most stubborn and depressing illnesses with limited progress in survival over the last decade. Although few advancements have been made in surgical and chemotherapeutical treatments for PC, only a fraction of patients have been cured. Many obstacles persist, and the overall diagnosis remains dismal for patients in different stages combined. The essential objective of PC research should be focused on more effective therapies with overcoming drug resistance and developing methods to target existing drugs to tumour cells should be prioritised as well. Future work will hopefully result in a better prognosis by early diagnosis and developing appropriate treatment regimens. It is envisioned that in the future surgery would be most likely adopted initially for debulking followed by other therapies such as the combinatorial approach. Combinatorial approaches based on individual phenotypic variations are expected to be adopted in clinical settings in future. To maximize the synergistic effect of the combination approach, the aptamer/siRNA and the chemotherapeutic drug must be internalised within the same cancer cell at a separately controlled time interval. Thus, their release profiles must be programmed efficiently. Also, the immune system activation, lack of understanding of biological processes and interactions, toxicity issues, challenges in manufacturing, clinical translation and marketing of these combinatorial therapies should be addressed in future. Though there are quite the hurdles in the realm of the combinatorial approach, we believe that this novel approach has the required potential to transform the narrative of PC, making it a reality sooner rather than later.

## Conclusion

PC is a deadly disease with overall low survival rates due to a lack of early diagnosis and availability of effective therapy. A five-year survival rate is only for about 10% of patients at all stages with patients dying majorly due to drug resistance. Gemcitabine is treated as the gold standard in PC for over decades; however, it is not curative alone. 80% of patients who are unsuitable for surgical procedures are treated via chemotherapy with or without adjuvant radiotherapy, thus tremendous research and effort have been made in the last few years to discover novel and effective therapies for the treatment of PC. Despite years of rigorous study, the development of efficient chemotherapies has been gradual, and there are no treatments available that are aimed at protecting of patient’s wellbeing. Patients with PC are in dire need of specific drugs or drug combinations that eliminate cancerous cells while minimizing adverse effects. An understanding of spatial metabolomic using sophisticated tool favoured the development of clinical therapies based on identified biomarkers. The combinatorial approach is based on the concomitant action of various therapeutic molecules to get the synergistic effect and thus enhance therapeutic efficacy. However, targeted approach with cocktail therapy to explicitly improve the clinical response. This review is primarily focused on the use of siRNA/aptamer in combination with other chemotherapeutics, which are beneficial in manipulating several pathways and effector proteins involved in tumour metastasis or drug resistance, thus can achieve the required therapeutic response. The combined chemotherapeutic and targeted approach using combination therapy and siRNA/Aptamer would increase patients’ survival rates and quality of life; however, the development and improvement are still minimal. We anticipate the combination therapy will provide patients with PC new optimism and confidence in treatment in future.

## Data Availability

Not applicable.
